# Supplementary material for: “Let’s Use This Mess to Our Advantage”: Calls to Action to Optimize School Nutrition Program beyond the Pandemic
Source: Int J Environ Res Public Health. 2022 Jun 22;19(13):7650. doi: 10.3390/ijerph19137650 (PMC9265650; doi:10.3390/ijerph19137650)
Supplement: Supplementary file 1 [file ijerph-19-07650-s001.zip › ijerph-1769346-supplementary.pdf]

Supplemental File S1. Interview guide.

Understanding reach and implementation of federal nutrition safety net programs for North Carolina families during the COVID-19 pandemic

Date of Interview: ________________

Subject ID Number: ________________

Interviewer's Name: ________________

Interview Start time (24 hour clock): ________________

Interview End time (24 hour clock): ________________

Section A: Introduction and brief study overview

Thank you for agreeing to participate in this study. The goal of this study is to understand the critical role of meal distribution programs in improving food access across the state and the innovative strategies being used to ensure families are fed during this time. The results of the interview in combination with the videos you provide will help to understand some of the challenges being faced at individual sites and the ways people are facing these challenges. During this interview we will ask questions about: your experiences in your role in the past few weeks, some of the challenges you have faced, and the strategies you have implemented. I would like to audio record the interview because I want to make sure I don't miss any of your comments. If you don't want the interview audio recorded, I will take detailed notes during the interview instead. The recording will be stored securely and eventually destroyed after we publish the study's findings.

Do I have your permission to record this interview?

□Yes **□No**

START AUDIO RECORDER

Section B: Pre-COVID and Site Selection

Okay great, I want to start by just seeing how things are going with your program. Tell me a little about what you're hearing and seeing.

Okay, let's back up a bit. I want to talk about what was "normal" with your program before all of this happened so that we can understand just how much has changed. Tell me about how your program operated before all this happening. Which programs were you operating before? *(e.g., National School Lunch Program, School Breakfast Program, Summer Food Service Program, At-Risk Afterschool Meals Program, Seamless Summer Option)*. And which ones have you been operating during the pandemic?

Probes:

1. In terms of food sources, preparation and distribution, what is different with your program than what you would usually do.
    a. Sources: order from distributor, local products, cost of food/supplies
    b. Preparation: food quality/nutritional value, scratch cooking
    c. Distribution: delivery methods, types of meals (breakfast, lunch, snack, supper), days per week meals provided, number of meals distributed at once
2. Thinking back to when schools first closed, tell me about the decision-making process for selecting distribution methods and sites. What factors did you consider?
    a. Factors: Staff, experience with summer, safety, "readiness" or preparedness, equity, ability to use school buses to transport meals
    b. School district leadership involvement

Section C: Processes/Challenges during COVID

Okay so you've told me a little bit about how the food sources, preparation, and distribution have been different than they used to. Is there anything else that comes to mind about how things are different now than they were before?

Probes:

1. Waivers: How much of what has been different, like what you described before, is related to the USDA waivers? Any additional thoughts on the process of using these waivers?
2. Programs: Are there other non-USDA programs in your district/area that are also feeding kids?
   a. Which programs, who is running, just during pandemic, how it affects meals
3. Staff. What does your staffing/volunteer situation like right now, compared to how it was before?
   a. Fewer staff willing/able to come in, fewer staff asked to come in, staff being asked to do different kinds of work, etc.
   b. Communication between sponsor and site staff/volunteers
   c. Who is staffing meal distribution (child nutrition staff, volunteers, principals, other school staff, bus drivers, etc.)
4. Longevity/Need: What are the things you've been thinking about in terms of keeping your program going, both as the pandemic continues as we return to normalcy?
   a. Funding, needs from local or state govt
   b. Biggest concerns for meeting needs of students
   c. More children in need of free meals with rising unemployment
5. Role of Partners
   a. New partners
   b. Existing partners taking on new roles

Tell me about some of the biggest challenges you've faced, and the strategies you have used to address them as the pandemic has evolved.

Tell me some things that you or your program have done that you are most proud of, or something impressive happening at any of your sites.

Section D: Looking to the future

Okay, I know it has been tough to think too far into the future, but let's try. First, is there anything about the process during the pandemic that could be useful to meet the needs of students when things are normal again?

Probes:

1. What have you learned about how we can be better prepared for future emergency school closures (as a nation, state, or district)? Is anyone in your district taking action?
2. What have you learned that can help us improve any part of the current process (procurement, preparation, distribution), not necessarily just during emergencies?

Second, imagine that 10 years from now, we are faced with another disaster like COVID-19. What would you say MUST CHANGE in the next 10 years to put your school foodservice program in the best position to respond? Dream big.

Section E: Summary

Okay, those are all my questions for you. [If time, sum up what was discussed]. Is there anything about this process that did not come up that you think is important for us to know?

Okay great, thank you so much for your time.

TURN OFF AUDIO RECORDER.
